# Imaging for risk stratification of sudden cardiac death

**DOI:** 10.1007/s00399-022-00884-6

**Published:** 2022-07-16

**Authors:** Pieter van der Bijl, Jeroen J. Bax

**Affiliations:** 1 Department of Cardiology, Heart Lung Centre, Leiden University Medical Centre, Albinusdreef 2, 2300 RC, Leiden, The Netherlands; 2 Turku Heart Centre, University of Turku and Turku University Hospital, Kiinamyllynkatu 4–8, FI-20520 Turku, Finland

**Keywords:** Implantable cardioverter-defibrillator, Left ventricular ejection fraction, SCD, Cardiac magnetic resonance, Ventricular arrhythmias, Implantierbarer Defibrillator, Linksventrikuläre Ejektionsfraktion, Plötzlicher Herztod, Kardiale Magnetresonanztomographie, Ventrikuläre Arrhythmien

## Abstract

Sudden cardiac death (SCD) can be effectively prevented with the use of implantable cardioverter-defibrillator (ICD). Current guidelines advocate an ICD for primary prevention in the presence of an left ventricular ejection fraction (LVEF) ≤ 35%. The majority of individuals that experience SCD, however, have an LVEF > 35%. Multimodality cardiac imaging has the ability to visualize the three factors responsible for arrhythmia-mediated SCD, namely substrate, trigger and modulator. Advances in cardiac imaging techniques have allowed improved SCD risk stratification, especially in the group of patients with an LVEF > 35%. However, clinical integration of cardiac imaging for SCD risk stratification will require more comparative data between modalities and parameters, as well as evidence of an impact on outcomes. The current review represents an update on the use of multimodality imaging techniques for SCD risk stratification.

## Abstract (de)

Der plötzliche Herztod („sudden cardiac death“ [SCD]) lässt sich mithilfe implantierbarer Defibrillatoren wirksam vermeiden. Aktuelle Leitlinien befürworten den Einsatz eines ICD zur Primärprävention bei Vorliegen einer linksventrikulären Ejektionsfraktion (LVEF) ≤ 35%. Die Mehrzahl der Personen mit SCD hat allerdings eine LVEF > 35%. Mit der multimodalen kardialen Bildgebung ist es möglich, die drei Faktoren zu visualisieren, die für einen arrhythmievermittelten SCD verantwortlich sind: Substrat, Auslöser und Modulator. Fortschritte in den Verfahren der kardialen Bildgebung erlauben eine bessere SCD-Risikostratifizierung, insbesondere in der Gruppe der Patienten mit einer LVEF > 35%. Allerdings verlangt die klinische Integration der kardialen Bildgebung in die SCD-Risikostratifizierung mehr vergleichende Studien zu Untersuchungsmethoden und Parametern sowie darüber hinaus den Nachweis eines Effekts auf die Behandlungsergebnisse. Die vorliegende Übersicht beschreibt den aktuellen Stand der Anwendung von Verfahren der multimodalen Bildgebung in der SCD-Risikostratifizierung.

## Introduction

Sudden cardiac death (SCD) is defined as an unexpected, terminal event occurring within 1 h of symptom onset when death was witnessed, or within 24 h of the deceased having been observed alive when death was an unwitnessed event. In most instances, SCD occurs as a result of significant, underlying structural heart disease, e.g. ischemic or non-ischemic cardiomyopathy or severe valvular heart disease. Electrophysiological abnormalities without macroscopic structural heart disease can also lead to SCD, but are far less common. The most frequent aetiology is ischemic heart disease, which accounts for 50–80% of SCD events [1]. SCD can be most effectively prevented with an implantable cardioverter-defibrillator (ICD), delivering antitachycardia pacing or defibrillation to terminate ventricular tachycardia or ventricular fibrillation. Deciding on ICD implantation for secondary prevention is usually straightforward—SCD which was interrupted by cardiopulmonary resuscitation or lethal arrhythmias which terminated spontaneously comprise indications for implantation of an ICD [2]. Establishing criteria for primary prevention ICD implantation, however, is more complex. Contemporary guidelines are based on an impaired left ventricular ejection fraction (LVEF) < 35%, measured on two-dimensional, transthoracic echocardiography [2]. Using LVEF in isolation, however, is neither sensitive nor specific, with up to 80% of individuals who experience SCD having a documented LVEF > 35% [3, 4]. The modest performance of LVEF alone in guiding SCD prediction may be attributed to various factors, including the presence of rhythms not amenable to ICD therapy (e.g. asystole or pulseless electrical activity) and the reduction of a complex pathophysiological process to LV systolic function. There is a clear need for improved risk stratification strategies to guide primary prevention ICD implantation, and while a variety of electrophysiological biomarkers have been described, the current review will focus on advances in the use of multimodality imaging to enhance SCD risk stratification.

## Visualization of SCD risk factors

**Fig. 1:**
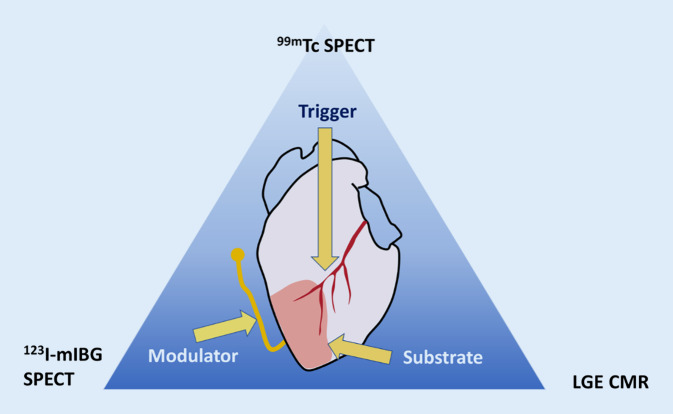
Coumel’s triangle of arrhythmogenesis. An illustrative example of Coumel’s triangle, where myocardial ischemia acts as a trigger, imposed on myocardial scar tissue as a substrate, modified by autonomic nervous system imbalance. Examples of modalities that can be used to image the different components of Coumel’s triangle are provided, i.e. late gadolinium enhancement cardiac magnetic resonance imaging (*LGE* *CMR*) for scar as a substrate, technetium 99m sestamibi single photon emission computed tomography (^*99m*^*Tc* *SPECT*) perfusion imaging for ischemia as a trigger and iodine-123 meta-iodobenzylguanidine (^*123*^*I‑mIBG*) SPECT for autonomic imbalance as a modulator

**Fig. 2:**
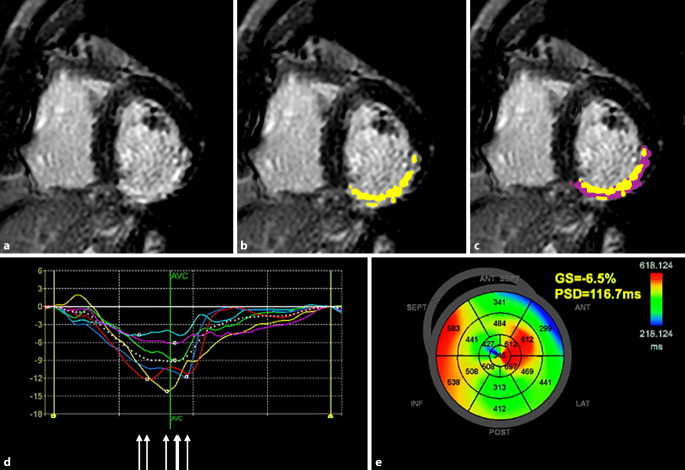
Multimodality imaging to assess the risk of sudden cardiac death (SCD) in ischemic cardiomyopathy. Short-axis, midventricular, late gadolinium enhancement (LGE) image of a patient with a previous transmural right coronary artery infarct on cardiac magnetic resonance (CMR) (**a**). Endocardial and epicardial borders were manually drawn (not shown), and the infarct size (LGE signal intensity ≥ 5 standard deviations of the remote myocardium) is shown in *yellow* (**b**). Grey zone (LGE signal intensity ≥ 2 standard deviations of the remote myocardium) is shown in *purple*, comprising 17.9% of the left ventricular myocardial mass (**c**). Mechanical dispersion (PSD) is calculated from strain-versus-time curves, derived from speckle tracking strain echocardiography (**d**). Peak longitudinal strain is non-uniformly timed (dispersed), as shown by *the* *white arrows*. Pathologically increased mechanical dispersion (116.7 ms) is displayed on a parametric map (**e**). Infarct and grey zone size on LGE CMR, as well as increased mechanical dispersion, are associated with SCD risk in ischemic cardiomyopathy. *ANT* anterior, *AVC* aortic valve closure, *GS* global strain, *INF* inferior, *LAT* lateral, *POST* posterior, *PSD* peak strain dispersion, *SEPT* septal

Life-threatening arrhythmias originate when a trigger (e.g. myocardial ischemia) is imposed on an arrhythmogenic substrate (e.g. ventricular scar tissue). The process can be further influenced by so-called modulating factors, e.g. autonomic nervous system dysfunction. This triad, comprising the factors responsible for arrhythmic SCD, is referred to as “Coumel’s triangle of arrhythmogenesis”, in honour of the eminent French electrophysiologist Philippe Coumel (Fig. 1). While the pathophysiology of post-infarct ventricular tachycardia is well understood (i.e. re-entry around scar tissue), the electrophysiological substrate in non-ischemic ventricular tachycardia is less well described [5]. Since scar tissue itself is electrically inert, ventricular tachycardias arise from the border zone (also called the “grey zone”), which is the transitional area between scar and normal myocardium (Fig. 2). This border zone represents an area of tissue heterogeneity, where non-uniform electrical conduction takes place and which is important for generating and sustaining ventricular tachycardias. Replacement scar can be imaged directly by late gadolinium enhancement (LGE) (Fig. 2) and indirectly with deformation imaging (reflecting the stiffness of scar), e.g. speckle tracking strain echocardiography or feature tracking cardiac magnetic resonance (CMR). Diffuse scar is reflected in elevated T1 values on parametric CMR mapping, although values can be influenced by aetiologies other than fibrosis, e.g. oedema and amyloid deposition. Progress in non-invasive techniques has allowed a shift from imaging scar tissue to the visualization of tissue heterogeneity, which can be quantified by measuring the size of the grey zone on LGE CMR, the mean absolute deviation of segmental pixel standard deviations on T1 mapping, mechanical dispersion (MD) on speckle tracking strain echocardiography (Fig. 2) or feature tracking CMR [5]. MD is defined as the standard deviation of the time of the onset of the QRS complex on the electrocardiogram (ECG) to peak myocardial deformation in 16 left ventricular segments, and reflects non-uniform electromechanical function due to underlying tissue heterogeneity, e.g. the presence of scar. It can also be influenced by electrical causes of dyssynchrony, e.g. a prolonged QT time (Fig. 2). While research has mostly focused on demonstrating the substrate of Coumel’s triangle, triggers can also be imaged, e.g. myocardial ischemia on stress perfusion CMR, pharmacologic stress echocardiography or nuclear perfusion single photon emission computed tomography (SPECT) or positron emission tomography (PET). Modulators (e.g. autonomic imbalance) can be visualized by nuclear innervation imaging, using radiolabelled analogues of noradrenaline (e.g. iodine-123 meta-iodobenzylguanidine [^123^I‑mIBG]), which compete with endogenous noradrenaline (the latter released by sympathetic nerves) for neuronal reuptake. Increased sympathetic tone causes a higher washout of the labelled noradrenaline analogues, which can be quantified with SPECT or PET. A summary of imaging modalities and techniques, stratified by the three components of Coumel’s triangle, is provided in Table 1.

**Table 1:** Summary of different imaging modalities and techniques used to image the components of Coumel’s triangle

*Substrate*	*Imaging modality*	*Technique*
Replacement scar	CMR	LGE
		Grey zone
Diffuse scar		T_1_ mapping
		ECV
Replacement and diffuse scar		Strain
		MD
	Echocardiography	Strain
		MD
*Trigger*	*Imaging modality*	*Technique*
Perfusion	Nuclear imaging	^99m^Tc SPECT
Inflammation		^18^F‑FDG PET
		^82^Rb PET
*Modulating factor*	*Imaging modality*	*Technique*
Denervation	Nuclear imaging	^123^I‑mIBG SPECT
		^11^C‑HED PET

^*11*^*C‑HED PET* ^11^C‑hydroxyephedrine positron emission tomography, *CMR* cardiac magnetic resonance, *ECV* extracellular volume, ^*18*^*F‑FDG PET* ^18^F‑labelled fluorodeoxyglucose positron emission tomography, ^*123*^*I‑mIBG SPECT* iodine-123 meta-iodobenzylguanidine single photon emission computed tomography, *LGE* late gadolinium enhancement, *MD* mechanical dispersion, ^*82*^*Rb PET* rubidium-82 positron emission tomography, ^*99m*^*Tc SPECT* technetium 99m sestamibi single photon emission computed tomography

## Ischemic cardiomyopathy

Patients with ischemic cardiomyopathy and an impaired LVEF demonstrated a survival benefit after ICD implantation in the Multicenter Automatic Defibrillator Trial (MADIT II) and the Sudden Cardiac Death in Heart Failure Trial (SCD-HeFT) [3, 4]. The majority of individuals who suffer from SCD, however, have an LVEF > 35%. Impaired LV global longitudinal strain (GLS) and increased LVMD (Fig. 2) on speckle tracking strain echocardiography have been independently associated with ventricular arrhythmias and SCD in patients with previous myocardial infarction, including those with an LVEF > 35% [6]. Visualization of LGE on CMR also represents a potential solution to the risk stratification of individuals with an LVEF > 35%, since both the presence and extent of LGE have been independently linked to SCD and ventricular arrhythmias in persons with ischemic cardiomyopathy, regardless of LVEF (Fig. 2; [7, 8]). Quantification of the grey zone on CMR, in addition to being independent of LVEF for predicting SCD, has been shown to be superior to the LGE burden (Fig. 2; [9, 10]). As an alternative to echocardiographic LVMD, SPECT has the ability to appraise LV dyssynchrony. In a study of 183 patients with severely impaired LVEF, those with greater SPECT-derived dyssynchrony experienced a higher frequency of ventricular arrhythmias [11]. Since no multivariable analysis was performed, no firm conclusion can be drawn regarding the additive value of dyssynchrony measured on SPECT for SCD risk estimation [11]. In a study of > 4500 patients with an LVEF > 35%, the extent of myocardial perfusion defects on SPECT was associated with SCD, demonstrating the potential value of imaging SCD triggers, in addition to the substrate [12]. Imaging the third limb of Coumel’s triangle, i.e. modulating factors of SCD, is currently the preserve of nuclear medicine. The AdreView Myocardial Imaging for Risk Evaluation in Heart Failure (ADMIRE-HF) trial investigated the role of ^123^I‑mIBG in predicting outcome in 961 patients with ischemic and non-ischemic cardiomyopathy and an LVEF < 35% [13]. The occurrence of SCD was associated with myocardial sympathetic dysfunction as part of a combined endpoint [13]. Similarly, in the Prediction of Arrhythmic Events with Positron Emission Tomography (PAREPET) trial, a greater burden of sympathetic denervation (visualized with ^11^C‑hydroxyephedrine PET) was associated with SCD in primary prevention ICD candidates with ischemic cardiomyopathy [14]. The Cardiovascular Magnetic Resonance Guided Management of Mild-Moderate Left Ventricular Systolic Dysfunction (CMR-GUIDE; NCT01918215) trial is a prospective, randomized study which is currently enrolling participants with an LVEF of 36–50%, i.e. persons who would not receive an ICD according to current guidelines [15]. In the Prediction of Arrhythmic Events With Positron Emission Tomography (PAREPET) II (NCT03493516) trial, the utility of ^18^F‑LMI1195 (a fluorinated noradrenaline analogue with a longer t_1/2_ than ^11^C‑labelled compounds, having the advantage of allowing delivery from a remote cyclotron) will be evaluated for the prediction of SCD in ischemic cardiomyopathy.

## Non-ischemic cardiomyopathy

Data on the utility of LVEF as a criterion for ICD implantation in non-ischemic cardiomyopathy are less consistent than for ischemic cardiomyopathy. While patients with an LVEF < 35% experienced a reduction in SCD in the Defibrillators in Non-Ischemic Cardiomyopathy Treatment Evaluation (DEFINITE) and SCD-HeFT trials, those with a similarly impaired LVEF did not show any improvement in survival in the Danish Study to Assess the Efficacy of ICDs in Patients with Non-Ischemic Systolic Heart Failure on Mortality (DANISH) [16–18]. Similar to ischemic cardiomyopathy, echocardiographic LVMD has been associated with SCD in patients with non-ischemic cardiomyopathy, independent of LVEF [19]. While the presence of LGE on CMR is also associated with SCD (independent of LVEF) and ventricular arrhythmias in persons with non-ischemic cardiomyopathy, there is little consensus on the extent and location required to accurately predict SCD [4, 20, 21]. Interestingly, a specific distribution of LGE, namely a ring-like pattern, was independently associated with ventricular arrhythmias in patients with dilated cardiomyopathy, and proved more robust than multifocal LGE [4]. No association was found between LGE and LVEF, which might partly explain the discordant results of ICD trials in non-ischemic cardiomyopathy [4]. Grey zone burden has been analysed in a population comprising ischaemic and non-ischaemic cardiomyopathy patients, suggesting that it also has a role to play in the SCD risk stratification of non-ischemic cardiomyopathy, although no subgroup analysis was performed [10]. A relation that is independent of LVEF has been established between native T1 mapping values and appropriate ICD therapy, SCD and ventricular arrhythmias in patients with non-ischemic cardiomyopathy [5, 22, 23]. Interestingly, in a prospective study of ICD recipients, native T1 mapping values were independently associated with SCD in non-ischemic cardiomyopathy, but not in ischemic cardiomyopathy [23]. T1 post-contrast mapping values are also predictive of ventricular tachycardia recurrence after catheter ablation [24]. In the International T1 Multicentre CMR Outcome Study (T1-CMR; NCT02407197), the utility of various CMR parameters, including LGE, ECV and T1 mapping, will be evaluated for SCD prediction in patients with non-ischemic cardiomyopathy.

## Hypertrophic cardiomyopathy

**Fig. 3:**
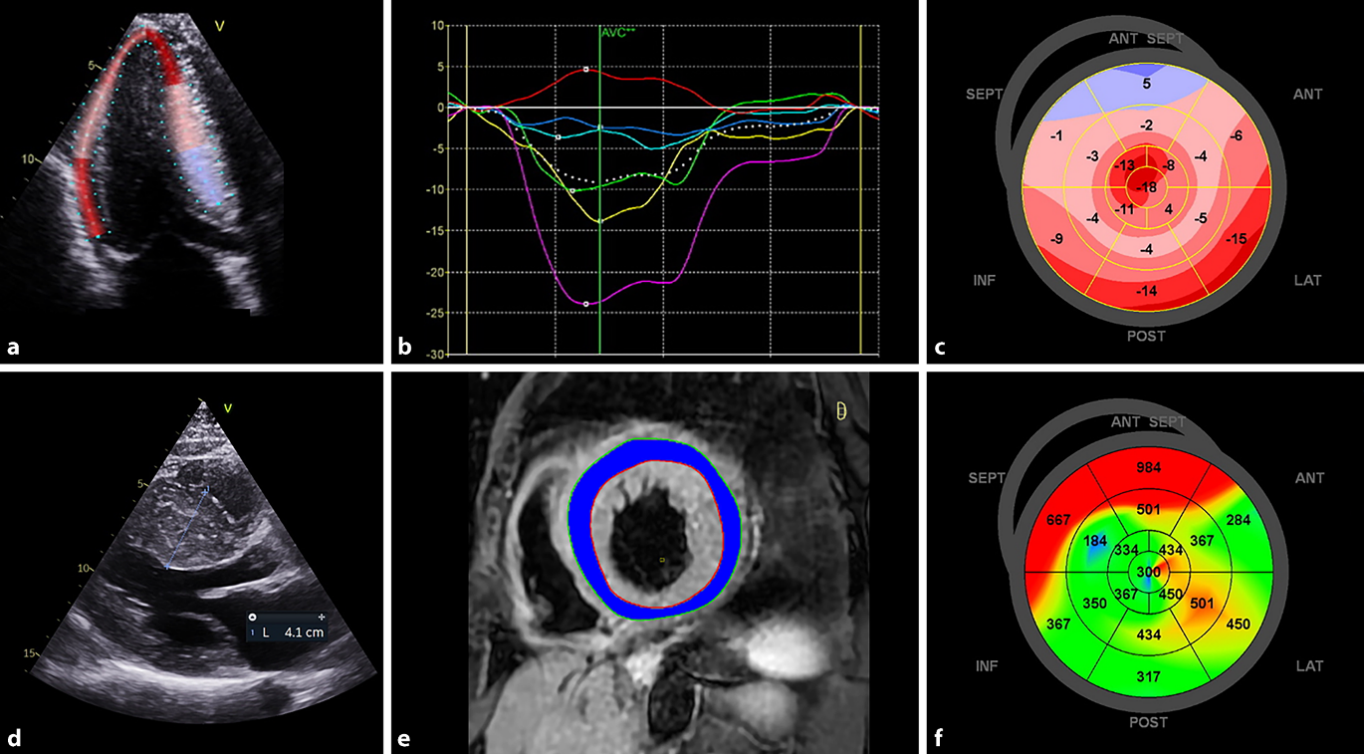
Multimodality imaging to assess the risk of sudden cardiac death (SCD) in hypertrophic cardiomyopathy (HCM). Longitudinal strain is measured with two-dimensional speckle-tracking echocardiography in an apical three-chamber view (**a**), generating segmental strain-versus-time curves (**b**). Impairment of left ventricular global longitudinal strain (−8.7%) is shown on a parametric map that demonstrates most severe impairment in the septum (**c**), which is the area of maximal wall thickening (**d**). T2-weighted short-tau inversion recovery short-axis image of the left ventricle, demonstrating an elevated T2 signal intensity ratio (3.3) when left ventricular myocardium (*blue*) is compared to skeletal muscle (*yellow*) (**e**). Mechanical dispersion (MD) of the left ventricle in the same patient as in **a**–**d**, displayed as a parametric map (**f**). Late activation of the septal segments (*orange/red*) results in an abnormally elevated MD of 183 ms (**f**). Impaired left ventricular global longitudinal strain, increased T2 signal intensity and increased MD are all associated with a higher SCD risk in HCM. *ANT* anterior, *AVC* aortic valve closure, *INF* inferior, *LAT* lateral, *POST* posterior, *SEPT* septal

Hypertrophic cardiomyopathy (HCM) is the most common cause of SCD in young adults, and the prevention of SCD is one of the primary management goals of this disease [25]. In a study of > 2400 individuals with HCM, the risk of appropriate ICD therapy was similar in patients with an LVEF of 35–40% and those with an LVEF < 35% [26]. A threshold of 35% can therefore not be directly transposed from ischemic cardiomyopathy to HCM for the purpose of SCD risk stratification. Impaired speckle tracking echocardiography-derived LV GLS, as well as an increased LVMD, have been linked to SCD risk and ventricular arrhythmias in persons with HCM (Fig. 3)—independent of LVEF [27, 28]. In studies demonstrating the prognostic value of LV GLS and LVMD, LVEF was not significantly associated with ventricular arrhythmias on univariable analysis, highlighting the limited value of using this parameter to risk stratify HCM patients [27, 28]. Both the presence and burden of LGE on CMR have been associated with ventricular arrhythmia and SCD risk in HCM—independent of LVEF. In a study of > 1200 persons with HCM, LGE extent remained independently associated with SCD when excluding individuals with an LVEF < 50% [29]. Use of LGE ≥ 15% of LV mass is recommended by the American College of Cardiology/American Heart Association HCM guideline as a marker of SCD risk [30–32]. Using the presence of LGE as a risk stratifier in HCM, however, is limited by the fact that 60–90% of patients with HCM have at least some degree of LGE [33]. While quantification of the LGE burden potentially circumvents this limitation, variation in LGE scanning sequences, LGE quantification and selection bias of studies make clinical implementation of LGE quantification challenging. In contrast to the US guideline, the European Society of Cardiology’s HCM guideline does not recommend the use of LGE for SCD risk stratification [32]. Elevated T2-weighted short-tau inversion recovery values on CMR have been associated with non-sustained ventricular tachycardia in a pilot study, likely reflecting myocardial oedema secondary to microvascular ischemia (Fig. 3; [33]). Native T1 mapping and extracellular volume (ECV) have also been correlated with SCD risk in HCM patients, and ECV was associated with SCD independent of LVEF [34, 35]. A multinational CMR study (NCT01915615) with the aim of investigating various CMR biomarkers in predicting SCD risk in HCM has completed enrolment, and results are expected in 2024 [36].

## Cardiac sarcoidosis

Similar to HCM, LVEF is limited in its ability to risk-stratify patients with sarcoidosis for SCD, since most who experience appropriate ICD therapy have an LVEF > 35% [37]. Impaired LV GLS was independently associated with ventricular arrhythmias and all-cause mortality in a study of 120 patients with cardiac sarcoidosis [38]. LVEF did not achieve significance for the primary endpoint in univariable analysis, emphasizing its limited value in this population [38]. The presence and extent of LGE on CMR are also associated with the risk of SCD, including those patients with LVEF > 35% [39]. PET imaging, demonstrating myocardial inflammation with perfusion-metabolic imaging (^82^Rb and ^18^F-[fluorodeoxyglucose] FDG), is another marker of ventricular arrhythmias in cardiac sarcoidosis patients—independent of LVEF [40]. Two studies, NCT03356756 and the Cardiac Sarcoidosis Multi-Center Prospective Cohort (CHASM-CS; NCT01477359), are enrolling patients for combined ^18^F‑FDG PET and CMR imaging and follow-up.

## Practical conclusion

Although the majority of individuals who experience SCD have an LVEF > 35%, the decision to implant an ICD for primary prevention remains predicated on an LVEF threshold of 35%. Modern cardiac imaging techniques can visualize different components of SCD arrhythmogenesis, namely the substrate, trigger and modulating factors. While most techniques are focused on the substrate, technical progress has allowed tissue heterogeneity to be imaged, rather than electrically inert scar. Multimodality cardiac imaging has demonstrated the ability to risk stratify patients with an LVEF > 35% effectively for the prediction of SCD. In order to integrate advanced cardiac imaging into routine practice for SCD risk stratification, future studies should address not only the relative merits of various imaging modalities and parameters to determine which have the highest utility, but also the lack of imaging-guided outcome data.

## Declarations

For this article no studies with human participants or animals were performed by any of the authors. All studies mentioned were in accordance with the ethical standards indicated in each case.
